# Self-adjuvanting Ebola virus-like particles that incorporate a cellular interferon-inducing domain

**DOI:** 10.21203/rs.3.rs-10832926/v1

**Published:** 2026-09-16

**Authors:** Naveen Thakur, Lin Wang, Tshidi Tsibane, Jesus A. Silvas, Viktoriya Borisevich, Rachel O’Toole, Jasmine Martinez, Thomas W. Geisbert, Robert W. Cross, Christopher F. Basler, JoAnn M. Tufariello

**Affiliations:** 1 Dept. Microbiology, Icahn School of Medicine at Mount Sinai, New York, NY, USA; 2 Center for Microbial Pathogenesis, Institute for Biomedical Sciences, Georgia State University, Atlanta, GA, USA; 3 Present address: Division of Urology, Department of Surgery, University of Colorado Anschutz, Aurora, CO, USA; 4 Present address: 7450^th^ Medical Operational Readiness Unit, US Army Medical Command, Aurora, CO, USA; 5 Galveston National Laboratory, University of Texas Medical Branch, Galveston, TX, USA; 6 Department of Microbiology and Immunology, University of Texas Medical Branch at Galveston, Galveston, TX, USA

## Abstract

Ebola virus-like particle (EBOV VLP) vaccines composed of glycoprotein (GP), matrix protein VP40, and nucleoprotein (NP) typically require multiple adjuvanted doses to elicit protection. To enhance immunogenicity and mimic live virus infection, retinoic acid-inducible gene I (RIG-I) N-terminal CARD domains (2CARD), which can trigger type I interferon (IFN) production, were fused to the NP C-terminal domain (NP_CT_). VLPs incorporating 2CARD-NP_CT_, GP and VP40 triggered a MAVS-dependent IFN response upon infection of A549 cells. In mice, a single immunization elicited robust antibody responses persisting for at least 20 weeks and provided complete protection against lethal challenge with mouse-adapted EBOV, whereas standard VLPs failed to do so. Furthermore, the system successfully incorporated *Mycobacterium tuberculosis* antigens via fusion to NP_CT_ or VP40. The findings demonstrate that incorporating 2CARD overcomes the low immunogenicity of standard VLPs. This modified EBOV VLP system serves as a potent, single-dose self-adjuvanting vaccine platform adaptable for various non-EBOV antigens.

## Introduction

The family *Filoviridae* includes Ebola virus (EBOV), Bundibugyo virus (BDBV), Marburg virus (MARV) and several other zoonotic pathogens that cause outbreaks of severe human disease and require biosafety level 4 (BSL4) containment for study. A 2005 MARV outbreak in Angola with a case fatality rate of ~90 percent^1,2^, the 2013-2016 West Africa EBOV outbreak, which caused >11,000 deaths^3,4^, and a BDBV outbreak that was identified in May 2026 in the Democratic Republic of Congo (DRC) and Uganda^5^ highlight the continued threat to global health posed by these viruses. Although FDA-approved antibody-based immunotherapy is available for the Zaire species of EBOV^6^, this is not true for other filovirus species, and approved small-molecule antivirals are also unavailable^7^. Given the scarcity of effective therapeutic agents, vaccines represent a critical strategy for combating filovirus outbreaks. Significant progress has been made advancing EBOV vaccines, whereas progress on vaccines for other filoviruses lags^7^.

A number of experimental EBOV vaccines with encouraging results in preclinical studies have advanced to testing and use in humans. The efficacy of rVSV-ZEBOV, a recombinant, replication-competent vesicular stomatitis virus-based candidate vaccine expressing a surface glycoprotein of Zaire EBOV, was assessed using a ring vaccination strategy targeting individuals at risk of exposure in Guinea and in Sierra Leone^8^. Administered as a single dose, the vaccine conferred 100% protection against EVD among individuals assigned to immediate vaccination^8^. A subsequent analysis of rVSV-ZEBOV vaccine efficacy in the Democratic Republic of Congo found that vaccination substantially reduced the case fatality risk (CFR) among patients with confirmed EVD, from 56.2% in unvaccinated patients to 17.6% for those vaccinated 10 days or more before symptom onset^9^. The rVSV-ZEBOV vaccine (Ervebo, Merck) was approved by the European Medicines Agency (EMA) and by the United States Food and Drug Administration (FDA) in 2019^10,11^. Regarding limitations, the vaccine provides limited cross-protection against other filovirus species in NHP models^12,13^.

Another strategy has employed replication-defective adenovirus vector-based Ebola vaccines. Vaccines based on adenovirus type 5 (Ad5), adenovirus type 26 (Ad26) and chimpanzee adenovirus type 3 (ChAd3) have been studied in clinical trials and found to be safe and immunogenic; although protective in non-human primate (NHP) models, efficacy in a human outbreak has not yet been demonstrated^14–23^. In some clinical trials, the adenovirus-vectored vaccines have been administered as part of heterologous prime-boost strategies^11,24^. A prime-boost approach involving Ad26.ZEBOV-GP (Zabdeno) and Mvabea (a Modified Vaccinia Ankara virus encoding EBOV GP, Sudan ebolavirus GP, Taï Forest ebolavirus NP and Marburg virus GP) was authorized for use in the European Union by the EMA^11,25^. This multi-dosing strategy may make this vaccine less suitable for an outbreak response setting^11,25^.

Virus-like particles (VLPs) are another promising, often-explored vaccine platform for a broad array of viruses, including for EBOV and MARV^26–34^. Advantages of VLPs include their relative safety, as they lack the viral genome and cannot replicate and cause viral disease, eliminating concerns about reversion of attenuating mutations and enabling use in the immunocompromised. Their close resemblance to authentic virus allows them to present antigens in their native state and elicit both humoral and cellular immune responses. They have proven efficacy for a variety of systems^35–38^. Further, VLPs can be engineered to present heterologous antigens^36^. The EBOV matrix protein, VP40, is able to bud from cells to form filamentous membrane-bound particles that resemble authentic EBOV^39–42^. Co-expression of the viral glycoprotein (GP) increases EBOV VLP production with GP present on the surface of the VLP membrane^42–45^. Co-expression of the viral nucleoprotein (NP) with VP40 also enhances budding and results in incorporation of NP into the VLPs^43,44^. VLPs formed by co-expression of VP40 and GP or VP40, GP and NP can protect mice, guinea pigs and non-human primates from lethal challenge^32,46–48^. EBOV VLPs stimulate dendritic cell (DC) cytokine responses and both B and T cell immune responses^32,47–51^.

Despite this efficacy, EBOV VLPs administered without adjuvant elicit relatively weak immune responses, with early studies showing minimal antibody responses following a single injection, modest responses after a single boost and more substantial responses after a third immunization^27,50^. Protection studies, whether in rodents or NHPs, have mostly involved multiple administrations of vaccine prior to challenge^46–49,51^, although a single administration of VLPs, which contained VP40, NP, GP and saponin derivative QS-21, included as adjuvant, protected 2 out of 3 immunized NHPs^32^. Complete protection was seen after boosting with a second dose^32^. EBOV VLPs can also mediate protection in a post-exposure prophylaxis setting, at least in mice, and this correlated with elicitation of type I interferon (IFN) responses^33,52^.

In this study, we sought to develop a VLP vaccine platform with substantially enhanced immunogenicity that better mimics virus infection, without the need for adjuvant. Our approach takes advantage of advances in our understanding of filovirus particle assembly to engineer ìenhancedî filoviral VLPs that efficiently incorporate a host-derived IFN signaling domain derived from retinoic acid-inducible gene I (RIG-I). When expressed in mammalian cells with intact RIG-I signaling pathways, the two N-terminal RIG-I CARD domains (2CARD) trigger production of type I IFN^53^. The C-terminal domain of EBOV nucleoprotein (NP_CT_) interacts with matrix protein VP40 and is critical for viral nucleocapsid incorporation into VLPs^43,54,55^. This suggested that NP_CT_ would be incorporated into EBOV VLPs. Therefore, 2CARD was fused to the EBOV NP_CT_. VLPs incorporating the 2CARD domain triggered a robust IFN response that is dependent upon MAVS, a key signaling protein downstream of RIG-I^56–59^. Vaccination with a single dose of these enhanced VLPs induced long-lived antibody responses in mice, and the mice were completely protected from challenge with a lethal dose of EBOV. In addition, the system successfully incorporated well-characterized *Mycobacterium tuberculosis* antigens via fusion to NP_CT_ or VP40; in this extension of the work, 2CARD was successfully fused to VP40. Therefore, the results support incorporation of 2CARD into VLPs to overcome low immunogenicity and show that 2CARD-containing EBOV VLPs can be adapted as a vaccine platform for non-EBOV antigens.

## Results

**Figure 1.**
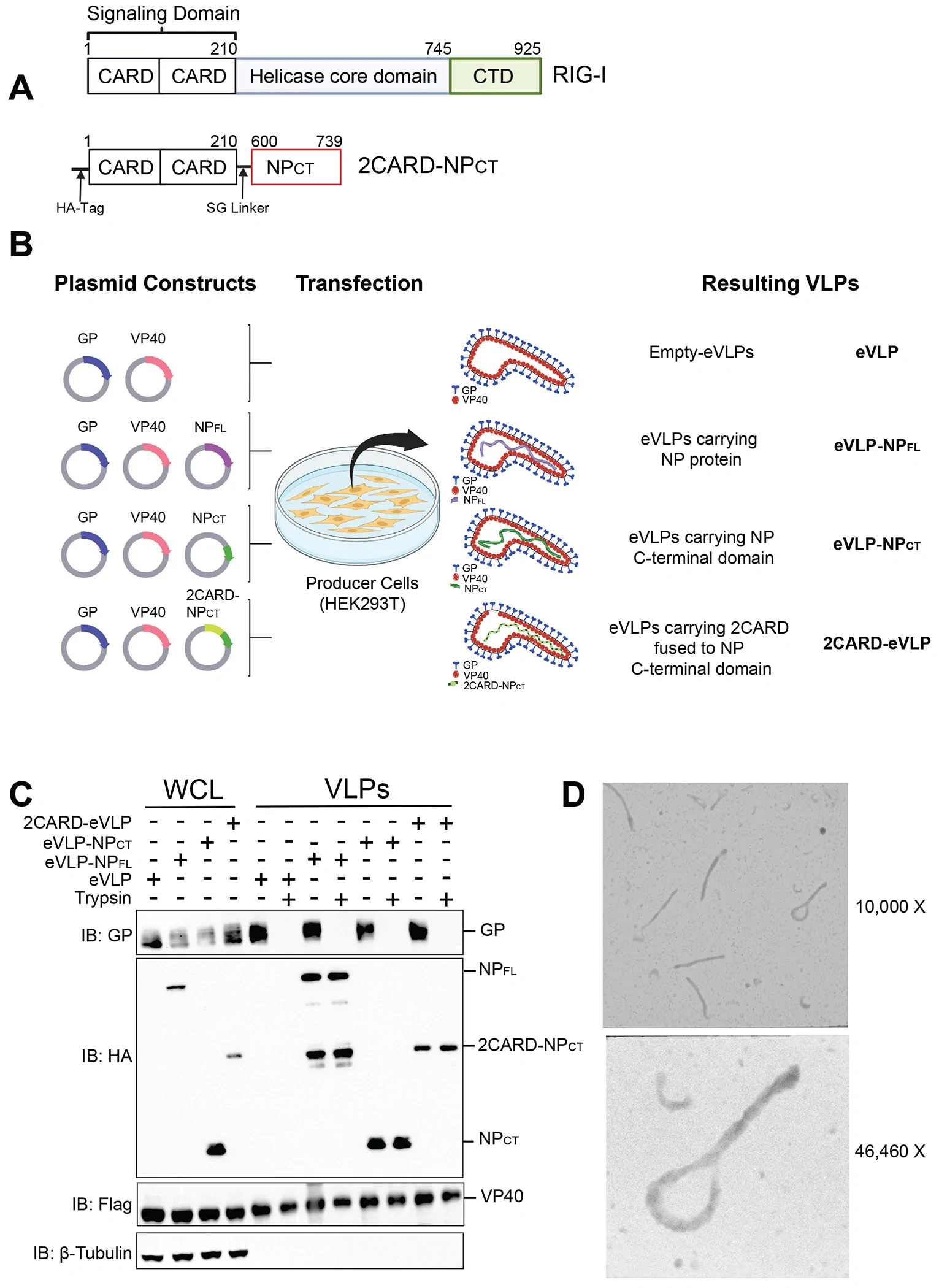
Incorporation of RIG-I 2CARD fusion proteins into eVLPs. A. Schematic of expression constructs showing HA-tagged RIG-I 2CARD (residues 1–210) fused to the C-terminal domain of Ebola virus NP (residues 600-739, NP_CT_). HA-tagged full-length NP (NP_FL_) and NP_CT_ alone served as controls. Adapted from an initial layout created with BioRender, Basler, C. (2026) https://BioRender.com/be84vs2. B. Strategy for production of Ebola virus–like particles (eVLPs). GP and VP40 were co-expressed to generate empty eVLPs, or together with HA-NP_FL_, HA-NP_CT_, or HA-2CARD-NP_CT_ to generate eVLP-NP_FL_, eVLP-NP_CT_, or 2CARD-eVLPs, respectively. Adapted from an initial layout created in BioRender. Tufariello, J. (2026) mhhtw2z. C. Western blot analysis of transfected whole cell lysates (WCL) and purified eVLPs. GP, Flag-VP40, and HA-tagged NP_FL_, NP_CT_, 2CARD-NP_CT_ constructs were expressed and incorporated as indicated. Trypsin treatment of purified VLPs removed GP but not VP40 or NP-derived proteins, consistent with surface localization of GP and internal incorporation of VP40 and NP constructs. β-tubulin served as loading control for the WCL. D. Representative transmission electron micrographs of purified 2CARD-eVLP preparations showing filamentous and curved morphologies characteristic of Ebola virus particles.

We sought a strategy to incorporate a constitutive IFN inducing domain into EBOV VLPs. The two amino-terminal RIG-I CARD domains, when expressed in mammalian cells, function as constitutive activators of the IFN-inducing RIG-I signaling pathway^53^. We reasoned that fusion of RIG-I 2CARD with the C-terminal region of EBOV NP might allow incorporation into EBOV VLPs. We therefore generated an expression plasmid in which an N-terminal HA tag followed by the first 210 amino acids of RIG-I was fused via a serine-glycine linker to the C-terminal domain of EBOV NP (NP_CT_) (Figure 1A). We also generated HA-NP_CT_ and HA-tagged NP full-length (HA-NP_FL_) constructs to serve as controls.

We used different combinations of expression plasmids to generate VLPs (Figure 1B). We co-expressed GP and VP40 to generate “empty” EBOV VLPs (eVLP). We also co-transfected GP, VP40 and either HA-NP_FL_, HA-NP_CT_ or HA-2CARD-NP_CT_ to produce eVLP-NP_FL_, eVLP-NP_CT_ and 2CARD-eVLPs. From the transfected cells, culture supernatants were collected and cell lysates were generated. After centrifugation of VLPs in the culture supernatants through a 20% sucrose cushion, the VLPs were resuspended and half of the material was then treated with trypsin. Western blotting of the lysates demonstrated expression of GP, Flag-VP40 and each of the HA-tagged NP constructs (Figure 1C). In the absence of trypsin treatment, GP and Flag-VP40 were detected at comparable levels for each of the VLP preparations. NP_FL_, NP_CT_ and 2CARD-NP_CT_ were also present in each of the eVLP preparations. Trypsin treatment eliminated GP, consistent with the protein being on the external surface of the eVLP membrane. However, Flag-VP40 and the NP constructs were unaffected by trypsin addition, consistent with these proteins being incorporated within the membrane of the VLPs (Figure 1C). Representative electron micrographs of the eVLP preparations demonstrated the presence of filamentous and curved particles, consistent with the expected morphology of EBOV VLPs^42,60^ (Figure 1D). The data demonstrate that NP_CT_ is incorporated into eVLPs and that fusion of a partner such as 2CARD to NP_CT_ is sufficient to mediate incorporation of the fusion.

**Figure 2.**
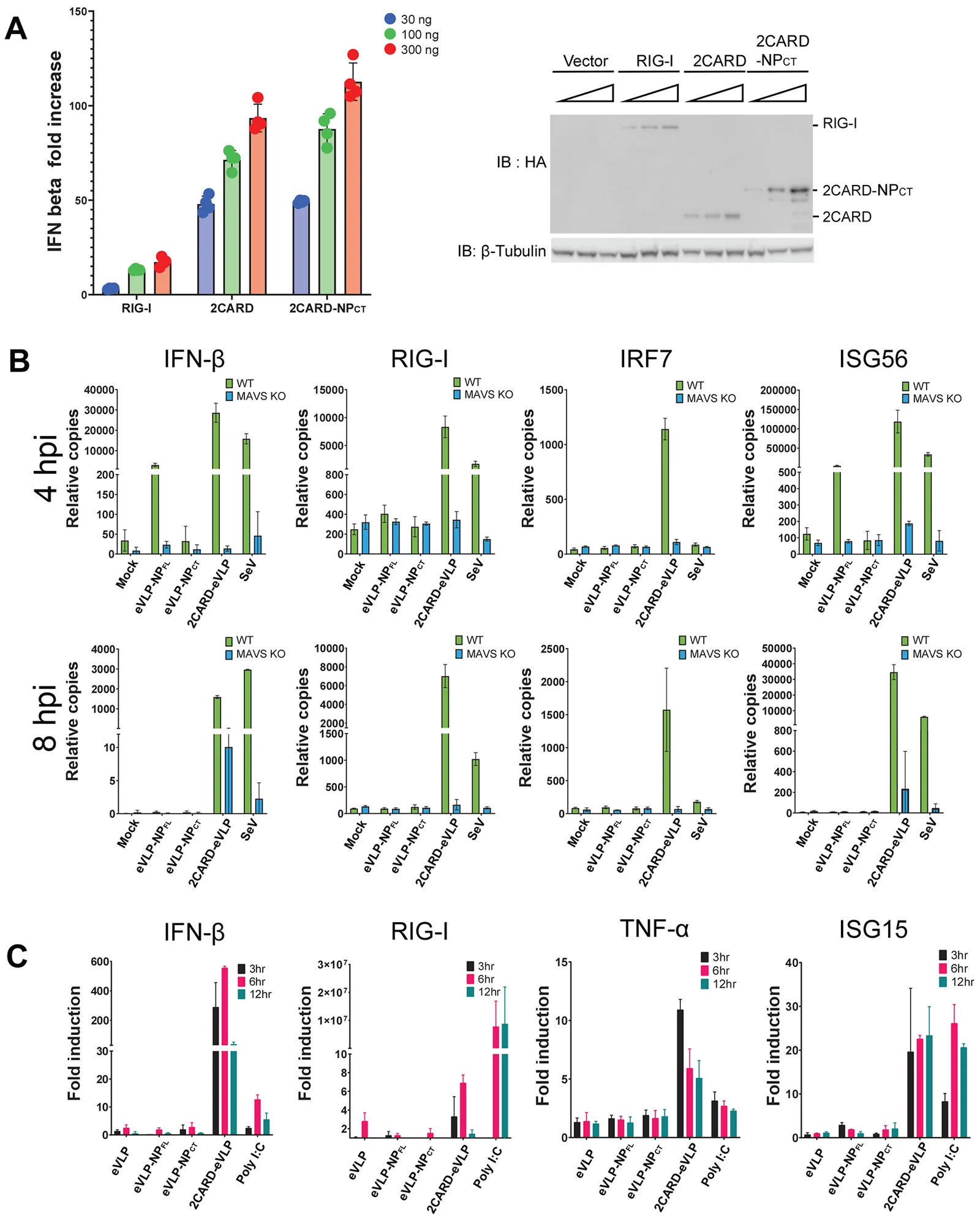
2CARD-NP_CT_ confers innate immune stimulatory activity to eVLPs. A. Reporter gene assay to assess activation of the IFN-β promoter. An IFN-β promoter-firefly luciferase reporter was co-transfected with a constitutively expressing Renilla luciferase expression plasmid and either empty vector or the indicated amounts of plasmids that express HA-tagged full-length RIG-I, RIG-I 2CARD or 2CARD fused to NPCT. Dual luciferase assays were performed at 24 hours post-transfection and firefly luciferase activity was normalized to Renilla luciferase activity. Fold increase was calculated relative to the empty vector control. Bars represent mean, symbols indicate individual data points and error bars indicate SD. Expression of the proteins was assessed by anti-HA Western blot with anti-β-tubulin serving as a loading control (right). Wedges indicate increasing amounts of transfected plasmid. B. RT–qPCR analysis of IFN-β, RIG-I, IRF7 and ISG56 mRNAs relative to β-actin mRNA at indicated time points post-infection in parental (green) and MAVS-deficient (blue) A549 cells mock infected, infected with the indicated eVLPs or infected with SeV, an activator of the RIG-I signaling. Bars represent mean and error bars indicate standard deviation (SD). RT–qPCR analysis of IFN-β, RIG-I, TNFα and ISG15 mRNAs in murine monocyte-derived dendritic cells at the indicated time points post-infection with the indicated eVLPs. Addition of poly I:C served as a positive control. Data are presented as fold-change relative to mock-infected controls, after normalization to β-actin. Bars represent mean and error bars indicate SD.

We used an IFN-β-luciferase reporter gene assay to assess the capacity of the 2CARD-NP_CT_ construct to induce an IFN response. Empty vector or plasmids that express full-length RIG-I, the RIG-I 2CARD domains and 2CARD fused to NP_CT_ were evaluated. Relative to empty vector, full-length RIG-I very modestly activated the IFN-β promoter, reflecting the fact that no RIG-I activating stimulus was provided. In contrast, the RIG-I 2CARD construct robustly activated reporter gene expression in a dose-dependent manner, reflecting the constitutive signaling that occurs when the CARD domains are separated from the regulatory helicase domain^53^. This capacity was retained when 2CARD was fused to NP_CT_ (Figure 2A). By western blot, full-length RIG-I and 2CARD were expressed to similar levels while 2CARD-NP_CT_ accumulated to modestly higher levels. Therefore, the 2CARD-NP_CT_ construct possesses the IFN inducing capacity of 2CARD alone.

We next determined whether 2CARD-eVLPs acquire the capacity to trigger IFN responses. MAVS is a critical signaling molecule that functions downstream of RIG-I^61^. Therefore, IFN responses triggered by 2CARD should be MAVS-dependent. To test this, we infected parental and MAVS knockout A549 cells with eVLPs generated with the different NP constructs and assessed IFN responses by quantitative RT-PCR. Mock treatment served as a negative control and infection with Sendai virus (SeV), a known RIG-I activator^62–65^, served as a positive control. Relative to the mock treatment, eVLP-NP_FL_ triggered a modest but transient increase in IFN-β and ISG56 mRNAs at 4 hours post-infection, but this was absent at the 8-hour time point. eVLP-NP_CT_ did not induce expression of the four genes assessed. In contrast, 2CARD-eVLPs triggered expression of each of the genes to levels comparable to SeV infection, and these increases were sustained at the 8 hour time point. The IFN responses triggered by 2CARD-eVLPs and by SeV were absent in the MAVS KO cells, confirming that the IFN response was triggered through the RIG-I pathway (Figure 2B). To assess the effects of the eVLP preparations on antigen presenting cells, we infected murine bone marrow-derived dendritic cells (BMDCs). For this experiment, we also included eVLPs generated by expressing only Flag-VP40 and GP, which is sufficient for formation of eVLPs^42,43,45^. In this experiment, the IFN inducer polyinosinic-polycytidylic acid (poly I:C) served as a positive control. The 2CARD-eVLPs triggered expression of IFN-β, the ISGs RIG-I and ISG15, as well as the cytokine TNFα, while the other eVLP preparations did not (Figure 2C). Cumulatively, these data demonstrate that eVLPs that incorporate 2CARD-NP_CT_ can trigger IFN responses via the RIG-I pathway, including in dendritic cells which are of particular relevance for triggering adaptive immune responses.

**Figure 3.**
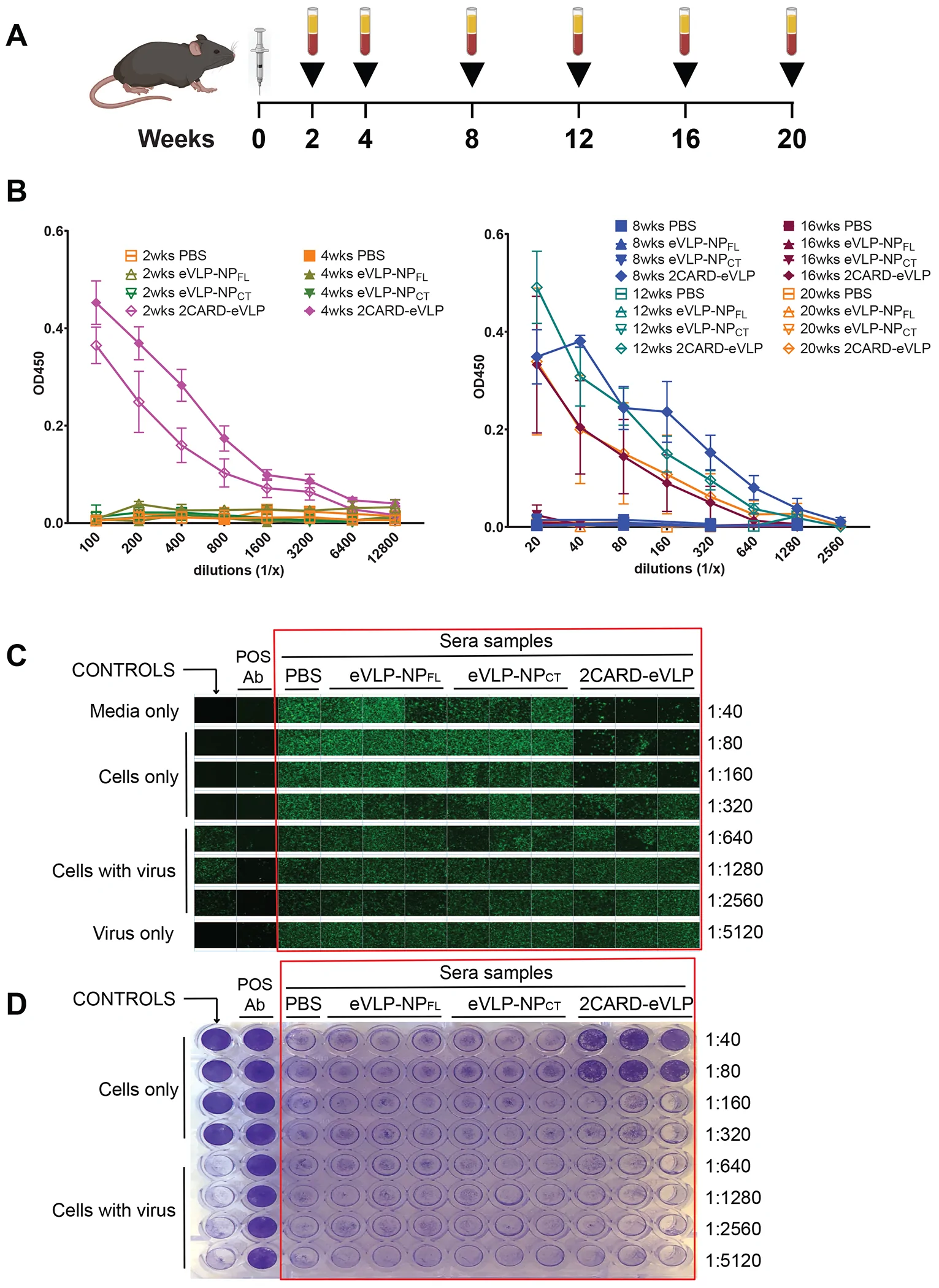
2CARD-eVLPs elicit neutralizing antibody responses. A. Immunization scheme and time points for assessment of GP-specific antibody responses following a single eVLP immunization. Adapted from an initial layout created in BioRender. Tufariello, J. (2026) https://BioRender.com/jkp6b7f. B. Anti-GP IgG responses measured by ELISA in mice immunized with PBS or with 10 μg eVLPs containing NP_FL_, NP_CT_, or 2CARD-NP_CT_. Robust and sustained GP-specific antibody responses were observed only in animals vaccinated with 2CARD-NP_CT_-containing eVLPs. Symbols represent mean and error bars indicate SD. n = 3 mice per group at each time point, except n = 1 for PBS control group at weeks 12, 16 and 20. C. Neutralization of VSV-GFP-GP by serial two-fold dilutions (indicated on the right) of 8 week sera from mice immunized with PBS or the indicated eVLPs. GFP expression was imaged with a Cytation 5 multi-mode reader (Biotek) as an indication of viral replication. POS Ab indicates wells in which VSV-GFP-GP was mixed with neutralizing monoclonal antibody 2G12 before addition to cells. Media only, cell culture medium in the absence of cells. Cells only, uninfected cells. Cells with virus, VSV-GFP-GP infection was performed in the absence of any antibody. Virus only, well with VSV-GFP-GP in cell culture medium in the absence of cells. D. Protection from VSV-GFP-GP-induced cytopathic effects by serial two-fold dilutions (indicated on the right) of 8 week sera from mice immunized with PBS or the indicated eVLPs. POS Ab indicates wells in which VSV-GFP-GP was mixed with neutralizing monoclonal antibody 2G12 before addition to cells. Cells only, uninfected cells. Cells with virus, VSV-GFP-GP infection was performed in the absence of any antibody. Cells were stained with crystal violet.

To determine whether eVLPs that incorporate 2CARD elicit enhanced adaptive immune responses, mice were immunized once with PBS or with eVLPs produced with NP_FL_, NP_CT_ or 2CARD-NP_CT_ and antibody responses to GP were assessed by ELISA (Figure 3A). Single administration of eVLPs with NP_FL_ or NP_CT_ elicited little anti-GP antibody. In contrast, 2CARD-eVLPs triggered production of anti-GP antibodies with levels sustained for at least 20 weeks post-immunization (Figure 3B). A vesicular stomatitis virus that expressed EBOV GP and GFP was used to determine whether neutralizing antibodies were produced. Controls included wells with no cells, wells with uninfected cells, wells with infected but untreated cells, and a well containing virus but no cells. In addition, wells infected in the presence of a previously described anti-EBOV GP neutralizing antibody 2G12 were included^66^. Sera from the 2CARD-eVLP-vaccinated animals were able to neutralize the virus at 1:80 to 1:160 dilutions as evidenced by reduced GFP expression (Figure 3C) and inhibition of cell killing (Figure 3D).

**Figure 4.**
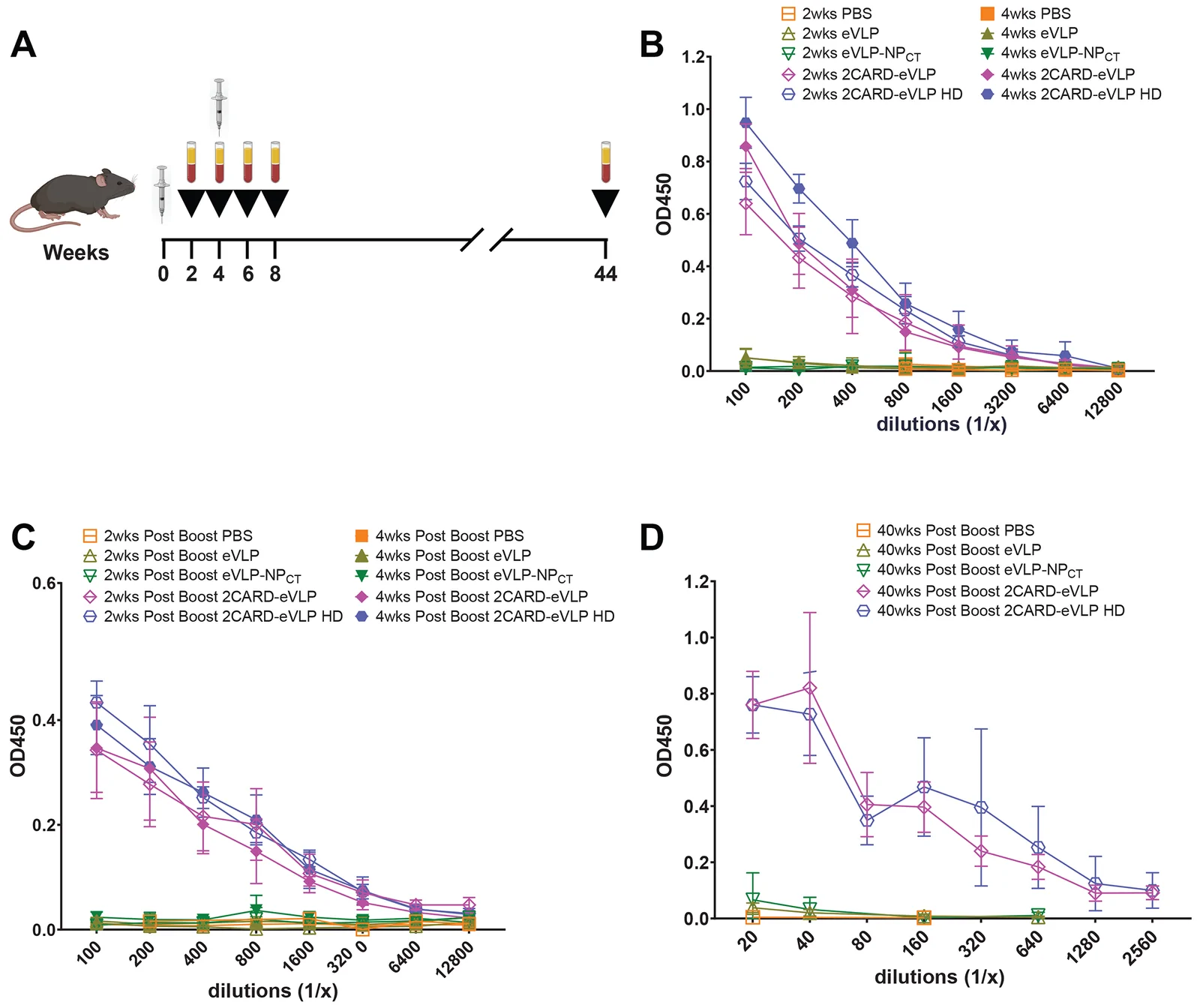
Prime–boost vaccination with 2CARD-eVLPs elicits robust, long-lived GP-specific antibody responses. A. Prime–boost immunization scheme comparing 10 μg eVLPs containing only VP40 and GP (eVLP), eVLP-NP_CT_, or 2CARD-eVLP. High dose (HD) for 2CARD-eVLP was 25 μg. Adapted from an initial layout created in BioRender. Tufariello, J. (2026) https://BioRender.com/jkp6b7f. B. Anti-GP IgG responses measured by ELISA at 2 and 4 weeks following priming. Symbols represent mean and error bars indicate SD. n = 5 mice per group. C. Anti-GP IgG responses measured by ELISA at 2 weeks and 4 weeks following booster immunization. Symbols represent mean and error bars indicate SD. n = 5 mice per group, except for PBS controls where n = 1. D. Long-term durability of GP-specific antibody responses measured by ELISA 40 weeks post-boost. Symbols represent mean and error bars indicate SD. n = 5 mice per group, except for PBS controls where n = 1.

The response to a prime-boost immunization approach was also assessed, comparing eVLP, eVLP-NP_FL_, eVLP-NP_CT_, and two doses of 2CARD-eVLPs (10 μg and 25 μg) (Figure 4A). As above, after an initial immunization, only the eVLPs with 2CARD-NP_CT_ elicited a detectable anti-GP antibody response at two and four weeks post immunization. The higher dose trended towards a modestly higher response (Figure 4B). Following a boost at four weeks, eVLPs with 2CARD-NP_CT_ again elicited the only significant anti-GP antibody responses (Figure 4C). The immunized animals were again bled forty weeks post boost. At this time point, animals primed and boosted with either of the two doses of 2CARD-eVLPs retained robust anti-GP titers (Figure 4D).

**Figure 5.**
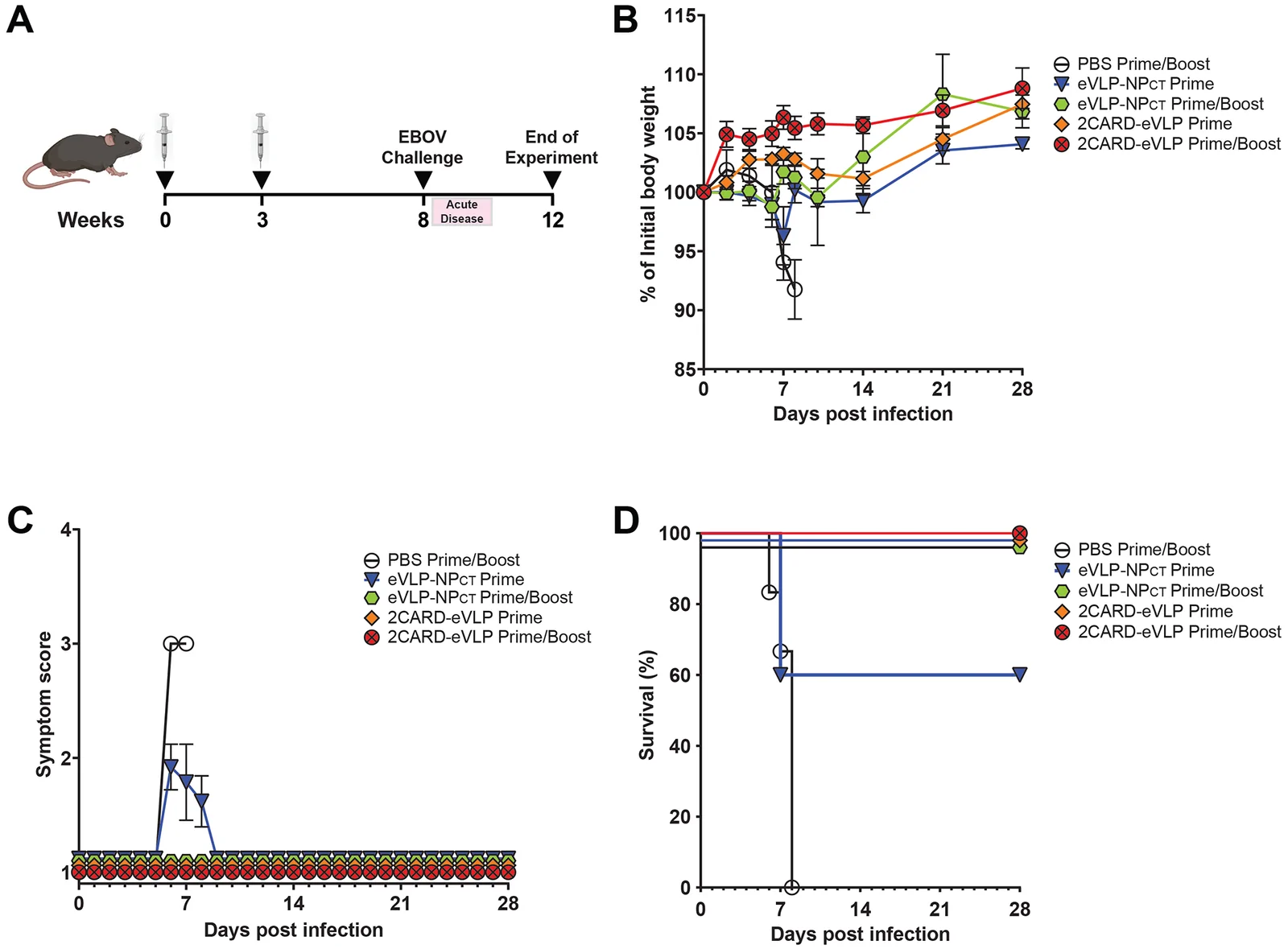
2CARD-eVLP vaccination confers protection against lethal Ebola virus challenge. A. Experimental design comparing single-dose (prime) and prime–boost immunization regimens with VLPs containing NP_CT_ and containing 2CARD-NP_CT_, followed by challenge with mouse-adapted Ebola virus. Period of acute disease is indicated. n = 6 mice for PBS group and 10 mice for remaining groups. Adapted from an initial layout created in BioRender. Tufariello, J. (2026) https://BioRender.com/kmt01pb. B. Body weight relative to starting weight (100%) for the indicated immunization groups. Symbols represent mean and error bars indicate standard error of the mean (SEM). C. Clinical scores following viral challenge for the indicated immunization groups. Symbols represent mean and error bars indicate SEM. D. Kaplan-Meier survival analysis following viral challenge for the indicated immunization groups.

The efficacy of the 2CARD-eVLP and eVLP-NP_CT_ vaccines was assessed in a mouse model of EBOV infection by comparing a single immunization (Prime) to a Prime/Boost regimen, with the boost administered three weeks after the initial immunization. Mice were challenged with a lethal dose of mouse-adapted EBOV five weeks after the boost. The animals were then followed for four more weeks (Figure 5A). After challenge, the PBS control group began to lose weight by day 4 and exhibited other clinical signs beginning on day 6, and all reached humane endpoints by day 8 (Figure 5B–D). Mice receiving a single dose of eVLP-NP_CT_ or two doses of eVLP-NP_CT_ in the prime-boost regimen exhibited on average modest weight loss at day 6. While four of ten eVLP-NP_CT_-immunized mice in the single immunization group developed additional clinical signs and succumbed, the eVLP-NP_CT_ prime-boost immunization animals regained their weight, did not develop additional symptoms and all survived the challenge. All of the animals that received immunization with 2CARD-eVLP, either as a single immunization or as a prime-boost regimen, survived and experienced no symptoms (Figure 5B–D). These data demonstrate that a single dose of 2CARD-eVLP elicits a protective immune response against EBOV challenge and is superior to a single dose of eVLPs also containing NP_CT_ but lacking the 2CARD domain.

**Figure 6.**
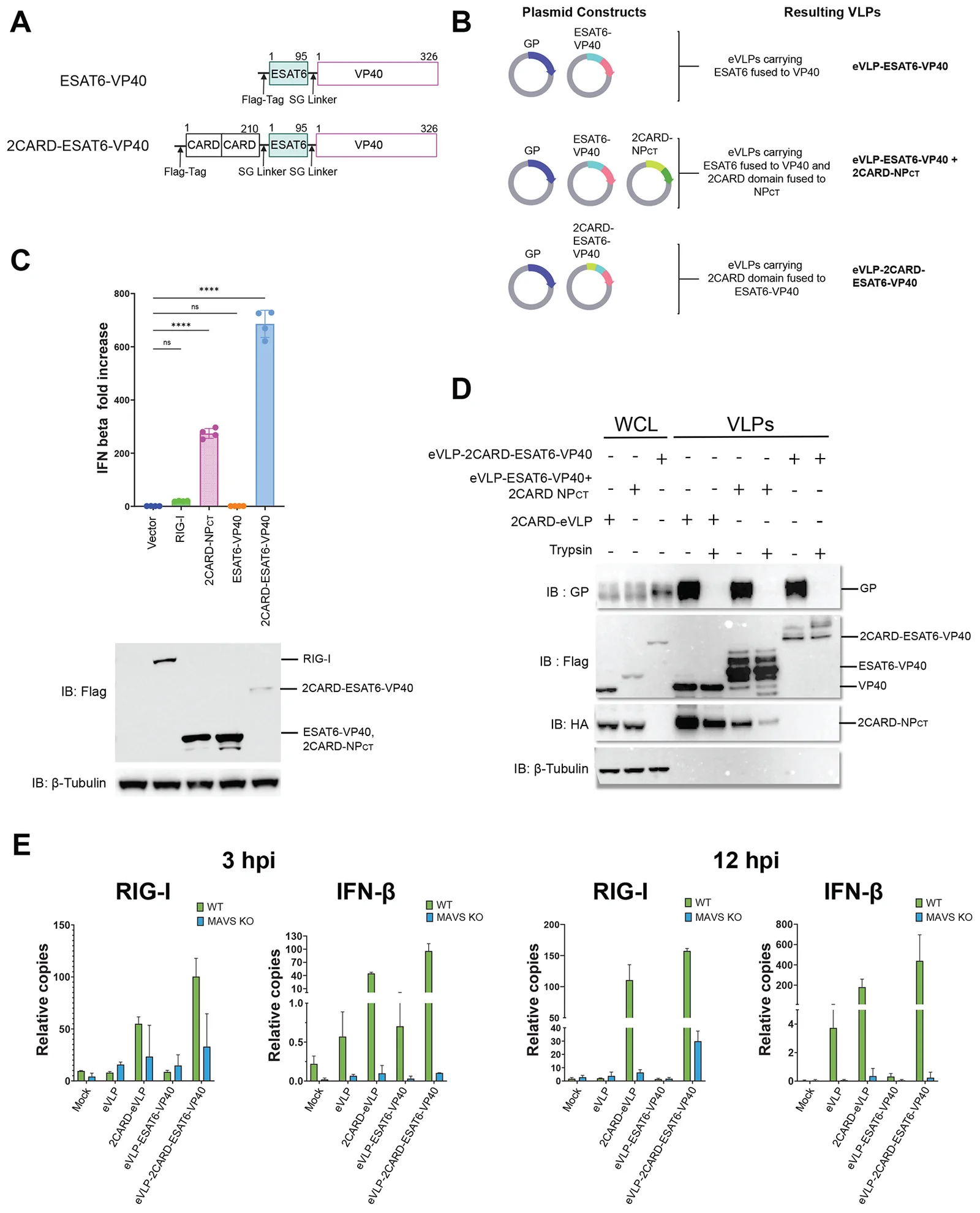
Successful incorporation into eVLPs of heterologous, *M. tuberculosis*-derived antigen ESAT-6 via fusion to VP40. A. Schematic of expression constructs showing Flag-tagged ESAT-6 fused to VP40, or ESAT-6 preceded by 2CARD domains and fused to VP40. Adapted from an initial layout created in BioRender. Basler, C. (2026) https://BioRender.com/yowbzyp. B. Strategy for eVLP production by co-expressing GP with ESAT6-VP40, ESAT6-VP40 plus 2CARD-NP_CT_, or 2CARD-ESAT6-VP40. Adapted from an initial layout created in BioRender. Tufariello, J. (2026) https://BioRender.com/rkly561. C. Reporter gene assay to assess activation of the IFN-β promoter following transfection with 300 ng of the indicated constructs in HEK293T cells. Fold increase was assessed relative to empty vector control (Vector). The assay was performed as in Figure 2A. Bars represent mean, symbols indicate individual data points and error bars indicate SD. Ordinary one-way ANOVA using Dunnettís multiple comparisons test was used to assess statistical significance. ****, p<0.0001; ns, not significant. Expression of the proteins was assessed by anti-Flag Western blot with anti-β-tubulin serving as a loading control. D. Immunoblot analysis of transfected whole cell lysates (WCL) and purified eVLPs. GP, Flag-VP40, -ESAT6-VP40, and -2CARD-ESAT6-VP40, and HA-2CARD-NP_CT_ constructs were expressed as indicated. Trypsin treatment of purified VLPs removed GP but not VP40- or NP-derived proteins, consistent with surface localization of GP and internal incorporation of VP40 and NP constructs. β-tubulin served as a loading control for the WCL. E. RT–qPCR analysis of IFN-β and RIG-I mRNA levels in parental (green) and MAVS-deficient (blue) A549 cells at 3 and 12 hours post-infection with the indicated eVLP preparations. Mock infection was also included as a control. Bars represent mean and error bars indicate SD.

In order to determine whether non-EBOV antigens can be incorporated into eVLPs that package the RIG-I 2CARD, two strategies were pursued. In the first, the well-characterized *Mycobacterium tuberculosis* antigen ESAT-6^67,68^ with an N-terminal Flag tag was fused to VP40, whereas a second construct included the N-terminal Flag tag followed by 2CARD domains and ESAT-6, also fused to VP40 (Figure 6A). These were used to produce eVLPs by co-expressing GP with ESAT6-VP40, by co-expressing GP with ESAT6-VP40 and 2CARD-NP_CT_ or by co-expressing GP with 2CARD-ESAT6-VP40 (Figure 6B). To evaluate the ability of the new constructs to induce IFN responses, we again used an IFN-β-luciferase reporter assay. HEK293T cells were transfected with empty vector or with plasmids expressing full-length RIG-I, 2CARD-NP_CT_, ESAT6-VP40, or 2CARD-ESAT6-VP40 (Figure 6C). As before, induction was minimal with full-length RIG-I, and as expected, no induction was apparent for the ESAT6-VP40 construct lacking 2CARD domains. As we observed previously (Figure 2A), the 2CARD-NP_CT_ construct robustly activated reporter gene expression, and this was also true for the 2CARD-ESAT6-VP40 construct (Figure 6C). Expression of each construct was assessed by Western blot. As expected, ESAT6-VP40 and 2CARD-NP_CT_ migrated with the same apparent molecular weight. Each of these constructs expressed to a higher level than 2CARD-ESAT6-VP40 (Figure 6C). The constructs were then evaluated for the capacity to produce eVLPs (Figure 6D). After transfections for eVLP production, each of the proteins was detectably expressed in whole cell lysates, although Flag-ESAT6-VP40 and Flag-2CARD-ESAT6-VP40 each accumulated to somewhat lower levels than Flag-VP40. When production of VLPs was assessed, ESAT6-VP40 was efficiently incorporated into VLPs, as was the co-transfected 2CARD-NP_CT_. 2CARD-ESAT6-VP40 was also incorporated into VLPs but at levels substantially lower than either VP40 or ESAT6-VP40. Due to the close co-migration of Flag-ESAT6-VP40 and Flag-2CARD-NP_CT_ observed when transfecting individual constructs (see Figure 6C), in the co-transfections for VLP production, we utilized HA-tagged 2CARD-NP_CT_ such that its expression could be established in the context of all VP40 constructs, including Flag-ESAT6-VP40. For each preparation of eVLPs, GP was sensitive to trypsin digestion, but the remaining proteins remained detectable, indicating incorporation of the other proteins within the eVLP membrane. It was notable, however, that trypsin addition reduced levels of 2CARD-NP_CT_ in the particles produced with ESAT6-VP40 (Figure 6D). Parental and MAVS knockout A549 cells were mock infected or infected with eVLP, 2CARD-eVLP, eVLP-ESAT6-VP40 or eVLP-2CARD-ESAT6-VP40 and IFN responses determined by assessing levels of RIG-I and IFN-β mRNAs (Figure 6E). In the parental A549 cells, 2CARD-eVLPs and eVLP-2CARD-ESAT6-VP40 induced expression of both genes at three and twelve hours post-infection (Figure 6E). eVLP and eVLP-ESAT6-VP40, which lack 2CARD, induced only a weak upregulation of IFN-β mRNA (Figure 6E). The upregulation detected in the parental cells were absent in the MAVS knockout cells, consistent with the IFN responses being activated via the RIG-I pathway.

**Figure 7.**
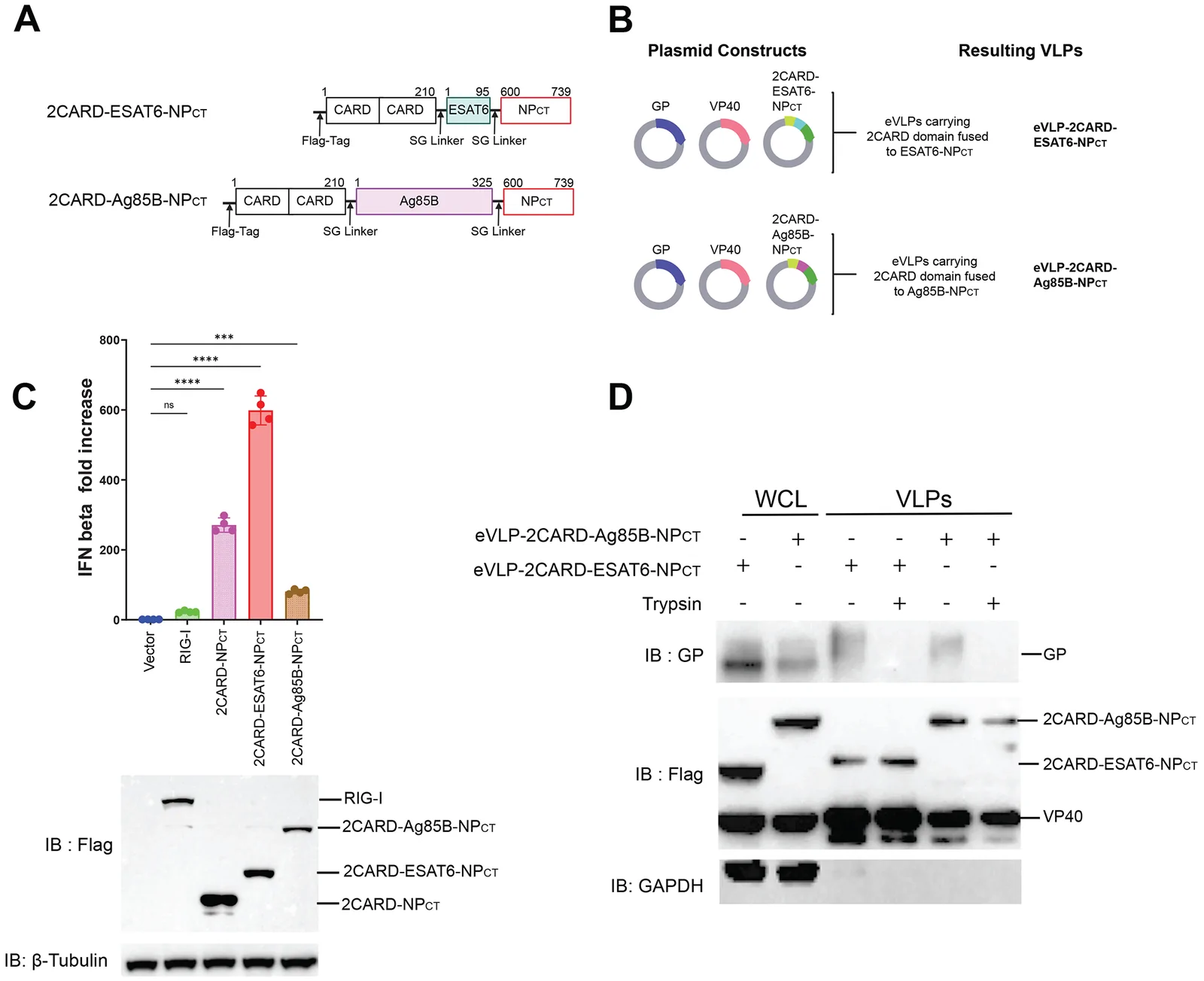
Successful incorporation into eVLPs of heterologous, M. tuberculosis-derived antigens ESAT-6 and Ag85B by insertion between 2CARD and NP_CT_. A. Schematic of constructs in which the M. tuberculosis-derived antigens ESAT-6 or Ag85B were positioned between 2CARD and NP_CT_. Adapted from an initial layout created in BioRender. Basler, C. (2026) https://BioRender.com/yowbzyp. B. Strategy for production of eVLPs incorporating 2CARD-ESAT6-NP_CT_ or 2CARD-Ag85B-NP_CT_ by co-expression with GP and VP40. Adapted from an initial layout created in BioRender. Tufariello, J. (2026) https://BioRender.com/qvd74t8. C. IFN-β luciferase reporter assay following transfection with 300 ng of the indicated constructs in HEK293T cells. The assay was performed as in Figure 2A. Fold increase was assessed relative to empty vector control (Vector). Bars represent mean, symbols indicate individual data points and error bars indicate SD. Ordinary one-way ANOVA using Dunnett’s multiple comparisons test was used to assess statistical significance. ****, p<0.0001; ***, p<0.001; ns, not significant. Expression of the proteins was assessed by anti-Flag Western blot with anti-β-tubulin serving as a loading control. D. Western blot analysis of transfected whole cell lysates (WCL) and the purified eVLPs. GP, Flag-VP40, Flag-2CARD-ESAT6-NP_CT_ and Flag-2CARD-Ag85B-NP_CT_ constructs were expressed and incorporated as indicated. Trypsin treatment of purified VLPs removed GP but not VP40 or NP-derived proteins, consistent with surface localization of GP and internal incorporation of VP40 and NP constructs. GAPDH served as loading control for the WCL.

Also explored was a third strategy, placing the foreign antigen between 2CARD and NP_CT_. For this, we inserted either ESAT-6 or another, larger *M. tuberculosis* antigen, antigen 85B (Ag85B)^68,69^ (Figure 7A). These were co-expressed with GP and VP40 to produce eVLP-2CARD-ESAT6-NP_CT_ and eVLP-2CARD-Ag85B-NP_CT_ (Figure 7B). Using an IFN-β-luciferase reporter assay in HEK293T cells, 2CARD-NP_CT_, 2CARD-ESAT6-NP_CT_, and 2CARD-Ag85B-NP_CT_ were all able to activate reporter gene expression, although the level of stimulation was lowest for 2CARD-Ag85B-NP_CT_ (Figure 7C). After transfections for eVLP production, each of the proteins was detectably expressed in whole cell lysates. eVLPs were formed that included 2CARD-ESAT6-NP_CT_ or 2CARD-Ag85B-NP_CT_, and these were incorporated within eVLPs, based on their resistance to trypsin (Figure 7D). Given that the individual constructs possessing 2CARD triggered IFN responses and that they are incorporated into eVLPs, we presumed that these eVLPs, like those described earlier, can trigger IFN responses.

**Figure 8.**
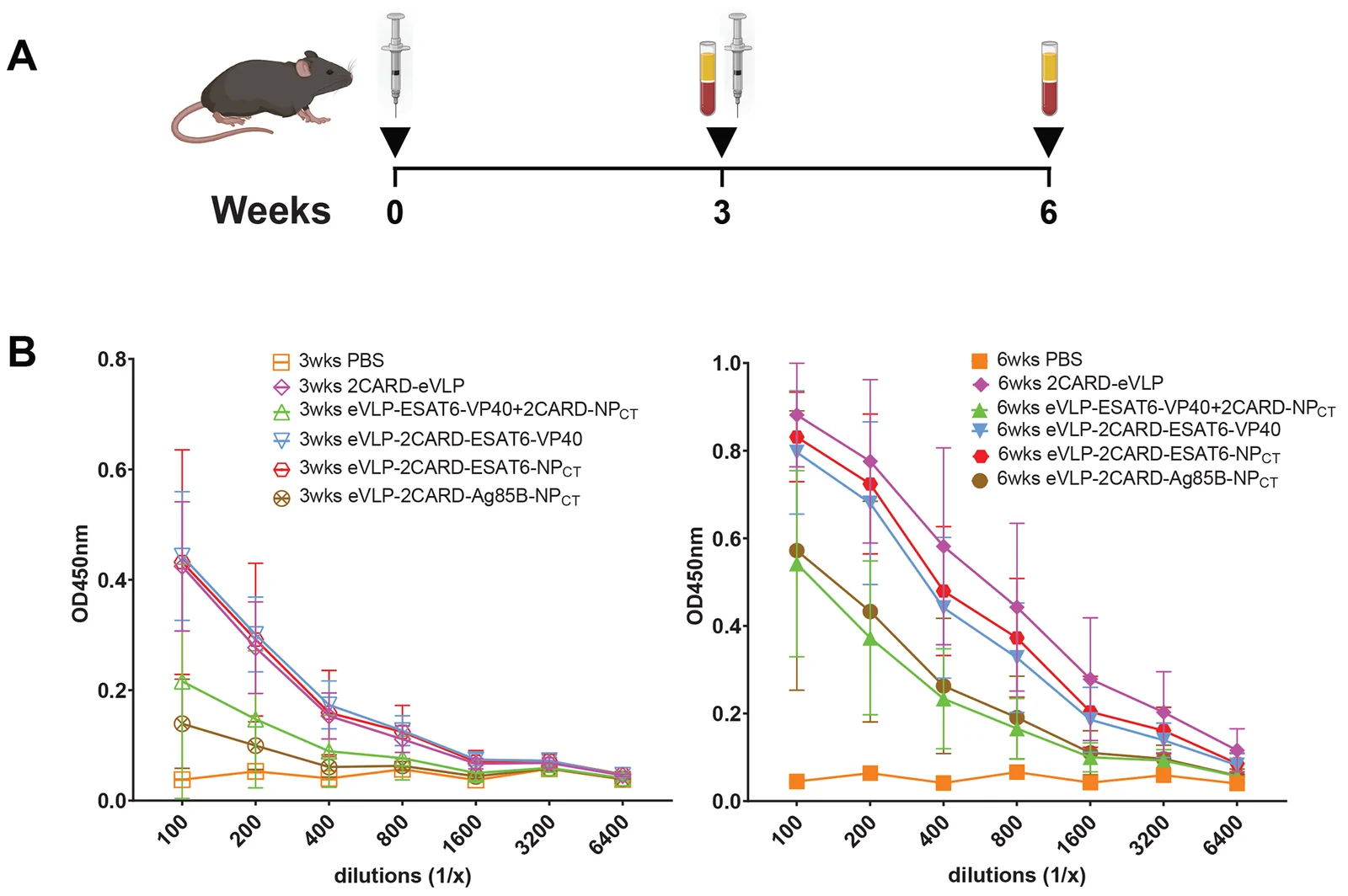
eVLPs incorporating M. tuberculosis antigens retain the capacity to elicit GP-specific antibody responses. A. Prime–boost immunization scheme comparing the indicated eVLPs. Adapted from an initial layout created in BioRender. Tufariello, J. (2026) https://BioRender.com/kmt01pb. B. Anti-GP IgG responses measured by ELISA at 3 weeks after priming and 6 weeks following priming / 3 weeks following boost. Mice were immunized with PBS or with 30 μg of the indicated eVLPs for both prime and boost. Symbols represent mean and error bars indicate SD. n = 3 mice per group.

Finally, the eVLPs with foreign antigens were tested for the capacity to elicit anti-GP responses. Mice were immunized, bled and boosted after three weeks and then bled again at six weeks (Figure 8A). After the first immunization, ELISAs demonstrated the strongest anti-GP responses to 2CARD-eVLP, eVLP-2CARD-ESAT6-VP40 and eVLP-2CARD-ESAT6-NP_CT_ (Figure 8B). Lesser responses were obtained with eVLP-ESAT6-VP40 + 2CARD-NP_CT_ and the Ag85B-containing VLP (eVLP-2CARD-Ag85B-NP_CT_). After boosting, mice vaccinated with 2CARD-eVLP, eVLP-2CARD-ESAT6-VP40 and eVLP-2CARD-ESAT6-NP_CT_ remained the strongest responders, but relative to these, responses to eVLP-ESAT6-VP40 + 2CARD-NP_CT_ and eVLP-2CARD-Ag85B-NP_CT_ increased (Figure 8B). These data demonstrate that it is possible to introduce foreign protein sequences into the eVLPs via fusions to NP_CT_ or to VP40 and that VP40 can accommodate fusions to 2CARD. However, the different responses to eVLP-2CARD-ESAT6-NP_CT_ versus eVLP-2CARD-Ag85B-NP_CT_ suggests that different foreign antigens may have varying impacts on the immunogenicity of this vaccine platform.

## Discussion

VLPs are a proven vaccine approach. Approved for use against human viral pathogens in various countries are VLP vaccines for hepatitis B virus (HBV), human papillomaviruses (HPV), hepatitis E (HEV) and Chikungunya virus^70–72^. While non-replicating, VLPs can present viral antigens in repetitive structures in a form that mimics authentic virus^38^. VLPs can also deliver viral proteins to antigen presenting cells and trigger both B cell and T cell responses^38^. EBOV VLPs have also been demonstrated to be promising, eliciting B and T cell responses and conferring protection from lethal challenge in rodents and non-human primates^26–38^. In our studies, we demonstrated that EBOV VLPs designed to incorporate the signaling 2CARD domains of the RNA-sensing RIG-I pattern recognition receptor exhibit enhanced immunogenicity as compared to EBOV VLPs that lack RIG-I 2CARD. A single immunization with the 2CARD VLPs elicited robust antibody responses persisting for at least 20 weeks and provided complete protection against lethal challenge with mouse-adapted EBOV, whereas standard VLPs failed to do so.

EBOV VLPs exhibit several features that are desirable for a vaccine. For example, EBOV VLPs display GP on their surface, and EBOV GP mediates tropism for APCs in vivo^73–75^. This tropism is likely mediated by GP interactions with cell surface attachment receptors. For example, C-type lectins DC-SIGN, DC-SIGNR/L-SIGN, LSECtin and human macrophage galactose- and N-acetylgalactosamine-specific C-type lectin (hMGL) bind to EBOV GP, to facilitate entry into cells, including dendritic cells and macrophages^76–80^. On activated DCs, the receptor Siglec-1 (a sialic acid-binding Ig-like lectin that binds viral envelope gangliosides) appears to play a particularly important role in attachment and entry^81^. Phosphatidylserine receptors also mediate EBOV attachment to facilitate infection^82–84^.

This tropism for APCs can promote T cell responses. EBOV infection of a mixed population of primary mouse DC subsets led to substantial T cell activation^85^. However, a number of studies found that EBOV infection fails to trigger the typical activation of human monocyte-derived dendritic cells^86–90^. This suppression of DC responses reflects the innate immune suppressing functions of the VP35 and VP24 proteins^88,91–93^. Unlike EBOV infection, eVLP vaccines do not express VP35 or VP24 in target cells. Perhaps because of this, EBOV VLPs trigger DC activation^50,94,95^. Mechanisms include activation of Toll-like receptor 4 and activation of NFκB and ERK1/2 MAP kinase signaling^95,96^. These effects are mediated, at least in part, by GP^95–97^. Activation of mouse DCs allows cross-presentation of antigen to prime CD8 T cells^85^. Consistent with this, EBOV VLP immunization triggers not only B cell but also T cell responses^32,47–51^.

Because past EBOV VLP studies often required multiple immunizations for robust immunity, we sought to augment the immunogenicity of EBOV VLPs. Our strategy takes advantage of the APC targeting and activating properties of eVLPs, while also introducing an innate immune activating component. Introduction into VLP-based vaccines of foreign components designed to enhance immunogenicity, including innate immune activating RNAs, has been described^98,99^. Our strategy was to incorporate RIG-I 2CARD in such a manner that it would be packaged into VLPs and be delivered intracellularly, where it would activate innate immune pathways. We chose RIG-I 2CARD because its expression potently activates type I IFN responses and therefore mimics responses elicited by natural viral infection^53^. Further, RIG-I agonists have been explored as adjuvants and as antivirals, and can promote adaptive immune responses^100–107^. Importantly, when we placed 2CARD N-terminal to fusion partners, the fusion protein retained the capacity to trigger IFN responses. Although a prior study demonstrated activation of the IFN responses by EBOV VLPs^33^, in our assays addition of 2CARD dramatically enhanced IFN responses relative to control eVLPs.

2CARD was also desirable because RIG-I functions as an intracellular pattern recognition receptor^108^. We reasoned that delivery of RIG-I 2CARD into the cell by VLPs would trigger activation of innate immune responses. Because activation of IFN response occurs intracellularly, RIG-I pathway activation should be restricted to cells that encounter VLPs and that are permissive for EBOV entry, particularly APCs. That the proteins within the VLPs would be delivered into the cell cytoplasm is supported by EBOV VLP entry assays that utilize a fusion of VP40 to β-lactamase^109–113^. In such assays, successful completion of the viral entry process is demonstrated by the delivery of the β-lactamase-VP40 fusion to the cell cytoplasm where β-lactamase cleaves the fluorescence resonance energy transfer (FRET)-based fluorescent substrate, CCF2, which has a cephalosporin core linking 7-hydroxycoumarin to fluorescein, leading to a shift in fluorescence^114,115^. Our hypothesis that 2CARD incorporation into VLPs would lead to IFN responses was validated. Supporting the expected mechanism of induction, IFN responses occurred when the 2CARD containing VLPs were added to cells with an intact RIG-I signaling pathway but abrogated in cells lacking MAVS, a key signaling molecule downstream of RIG-I^61^.

Our initial choice to fuse 2CARD with NP_CT_ was based on the demonstrated interaction of NP_CT_ with VP40 and because the interaction was needed for incorporation of viral nucleocapsids into VLPs^43,54,55^. Our data demonstrated that NP_CT_ is sufficient for VLP incorporation, and fusion of 2CARD to NP_CT_ did not substantially alter efficiency of incorporation as compared to NP_CT_ alone. We were also able to incorporate *M. tuberculosis* ESAT-6 or Ag85B between 2CARD and NP_CT_ and retain incorporation into VLPs. This suggests that the NP_CT_ fusion strategy is a flexible approach that allows incorporation of foreign sequences into EBOV VLPs.

We later fused 2CARD and *M. tuberculosis* antigens to the N-terminus of VP40. The rationale for the VP40 fusions was based on prior demonstrations that expression of VP40 alone is sufficient to drive budding of VLPs, although budding is enhanced by co-expression of other viral proteins including GP and NP^39–42^. In addition, VP40s fused at their N-terminus to foreign proteins, including β-lactamase, GFP, firefly luciferase, Nanoluc luciferase and BioID2, retain the capacity to bud from cells as VLPs^42–45,109,116–123^. Consistent with the capacity of VP40 to bud with foreign sequences fused to its N-terminus, our ESAT6-VP40 fusion protein was robustly released from transfected cells at levels that exceeded the Flag-tagged VP40 used as a control. Interestingly, ESAT6-VP40 incorporated into VLPs produced a laddering pattern on Western blots. The basis for the various forms detected requires further investigation. By comparison, addition of 2CARD to the N-terminus of ESAT6-VP40 resulted in less expression and correspondingly reduced release as VLPs. Whether the increased size of 2CARD-ESAT6-VP40 explains the reduced efficiency or whether specific sequences impair VP40 budding, remains to be determined.

The successful incorporation of additional non-viral sequences was performed as a proof of platform modularity. The successful incorporation of *M. tuberculosis* antigens ESAT-6 and Ag85B demonstrates the flexibility and expands the potential utility of the EBOV VLP system. Our strategy results in the bacterial antigens being incorporated within the VLP. This was demonstrated by the resistance of these proteins to degradation by trypsin. Therefore, this strategy should deliver the *M. tuberculosis* antigens intracellularly, enabling presentation on MHC molecules. However, we have not yet determined whether or how well the VLPs elicit T cell responses. There are other examples where non-viral antigens have been incorporated into VLP-based vaccines. For example, the RTS,S/AS01 and the R21/Matrix-M vaccines present malaria circumsporozoite protein on the surface of Hepatitis B surface antigen (HBsAg)-based VLPs^124^. *Helicobacter pylori* KatA has also been presented on HBsAg-based VLPs^125^. A glycosylphosphatidylinositol-anchored *Brucella* BCSP31 protein was incorporated into VLPs when co-expressed with the Newcastle disease virus matrix protein and elicited antibody and T cell responses in mice^126^.

Future studies should evaluate different routes of inoculation. The intraperitoneal route used in the present studies was chosen because it is straightforward and reliable in mice. However, intramuscular and subcutaneous routes warrant evaluation. Oral administration also deserves consideration, as immunization by this route with VLPs produced by co-expressing GP, VP40 and NP elicited anti-EBOV antibody responses and conferred partial protection of type I IFN receptor-deficient (IFNAR^−^/^−^) mice from EBOV challenge^127^. Other methods to produce 2CARD-eVLPs should also be explored. For these studies, we relied on transient co-transfection of HEK293T cells with expression plasmids for each component of the vaccine. This resulted in consistent production of VLPs that could be concentrated by centrifugation through a sucrose cushion. A concern with this approach is that 2CARD expression in this cell line can trigger IFN responses. This may impact expression. Insect cell systems have previously been used to produce EBOV VLPs^32,51,128^. This may offer an advantage in that insect cells do not produce RIG-I or MAVS and lack a type I interferon response^129^. It would therefore be of interest to determine whether yield of the 2CARD VLPs would be enhanced in insect cells. Alternatively, the VLP components could be encoded in a nucleic acid-based format, such as plasmid DNA or mRNA. Utilizing these platforms should lead to *in vivo* production of VLPs. Given that the strategy outlined here works for EBOV, it is likely to be effective for other filoviruses. Future research should evaluate options for incorporating 2CARD into VLP vaccines for additional viral families.

## Materials and Methods

### Cell Culture and viruses

The HEK293T, A549 WT, A549 MAVS knockout and Vero cell lines were grown at 37°, 5% CO_2_ in Dulbecco’s Modified Eagle Medium (DMEM) containing glucose and glutamine (Corning 10-013-CV) supplemented with 10% fetal bovine serum and penicillin/streptomycin. The A549 MAVS knockout cell line was kindly provided by Susan R. Weiss, University of Pennsylvania^130^. Recombinant vesicular stomatitis virus (VSV) expressing enhanced green fluorescent protein (eGFP) and EBOV GP (VSV-GFP-GP) was kindly provided by Kartik Chandran (Albert Einstein College of Medicine)^131^ and was amplified on Vero E6 cells. Mouse-adapted Ebola virus (Ebola virus M. musculus/COD/1976/Mayinga-CDC-808012)^132^ was kindly provided Dr. Alex Freiberg^133^ and passaged once on Vero cells.

### Construction of Plasmids for Ebola VLP Production

Plasmids EBOV GP, NP_FL_ and VP40 have been previously described^95,134^ . The codon-optimized 2CARD region (amino acids 1–210) from the RIG-I gene was fused to NP_CT_, ESAT-6 and Ag85B from *M. tuberculosis* H37Rv via a flexible SG linker. ESAT-6 and Ag85B were fused via a flexible SG linker to NP_CT_. ESAT-6 and 2CARD-ESAT-6 were fused to VP40 in a similar manner. Codon optimized constructs were synthesized with NotI and XhoI restriction sites at the N- and C-termini in a pUC57 backbone (GenScript). For expression, the fragments were subcloned into the pCAGGS vector with sequences encoding an N-terminal Flag or HA epitope tag. All plasmid inserts were verified by sequencing.

### Transient transfection and VLP purification

Transient transfections were performed using Lipofectamine 2000 (Invitrogen 11668500) according to the manufacturer’s instructions. For 6-well plates, 1.0 × 10^6^ cells were seeded per well. Expression was assessed 48 h post-transfection by Western blot.

For VLP production, 6.0 × 10^6^ cells were seeded in 150-mm dishes (Fisher Scientific 12-556-003). After 48 h incubation, transfected cell culture supernatants were collected and clarified by slow speed centrifugation before ultracentrifugation through a 20% (wt/vol) sucrose cushion at 25,000 ×*g* for 3h at 4 °C. The resulting pellet, which was not visible to the naked eye, was resuspended in NTE buffer (10 mM Tris-HCl pH 7.4, 100 mM NaCl, 1 mM EDTA). VLPs were stored at −80 ° C until use.

### Electron Microscopy

Purified eVLP samples were visualized by electron microscopy. Briefly, 10 μl of purified eVLPs were allowed to adsorb onto nickel grids for 10 min. After initial adsorption, grids were washed with ultrapure water 3 times for 1 min each time and fixed with 2% aqueous glutaraldehyde for 10 min. Following fixation, the grids were then washed 3 times with ultrapure water. Lastly, the grids were stained with UranyLess electron microscopy stain for 15 s, blotted dry and visualized on a LEO 906 TEM at 80kV.

### Immunoblotting

Cells were lysed in NP-40 lysis buffer (50 mM Tris-HCl pH 8, 280 mM NaCl, 1% NP-40, 0.2 mM EDTA, 2 mM EGTA) supplemented with protease inhibitor cocktail (Sigma-Aldrich 11836170001). Lysates were cleared at 16,000 ×*g* for 10 min at 4 °C, denatured in Laemmli buffer and resolved by Invitrogen Bolt 4% to 12%, Bis-Tris gels (NW04120BOX). Proteins were transferred to PVDF membranes (Sigma-Aldrich 03010040001), blocked in 5% milk and probed with primary antibodies against Flag (Rabbit Sigma-Aldrich F7425, Mouse Sigma-Aldrich F3165) or HA (Rabbit Invitrogen 71-5500, Mouse Sigma-Aldrich H3663), EBOV GP (4F3) (IBT Bioservices 0201-020), GAPDH (Mouse Sigma-Aldrich G8795) and β-tubulin (Mouse Sigma-Aldrich T8328). HRP-conjugated secondary antibodies (Cell Signaling Technology 7076S, 7074S) were used, and signals were detected by chemiluminescence (Perkin Elmer NEL105001EA) on a ChemiDoc MP Imaging System (Bio-Rad).

### IFN-β promoter–luciferase reporter assays

Cells were co-transfected with an IFN-β promoter-firefly luciferase reporter plasmid pIFN-β-Luc, and a constitutively expressing *Renilla* luciferase plasmid pRL-TK (Promega), together with the expression constructs as specified. Luciferase activity was measured 18–24 h post-stimulation using the Dual-Luciferase Reporter Assay System (Promega E1960) on an Agilent BioTek Cytation C10 Confocal Imaging Reader. Firefly luciferase values were normalized to *Renilla* luciferase values. Data are presented as fold change relative to the vector control. Each transfection was performed in triplicate, and each experiment was repeated at least twice, yielding similar results.

### qRT–PCR

Total RNA was extracted using the Direct-zol RNA Kit (Zymo Research, R2052) and reverse-transcribed into cDNA with the SuperScript IV VILO Master Mix incorporating ezDNase Enzyme (Thermo Fisher Scientific, catalog no. 11766050). Quantitative real-time PCR (qPCR) was performed using PerfeCTa SYBR Green FastMix (catalog no. 101414-272) on a Bio-Rad CFX Opus 96 detection system. The forward (F) and reverse (R) primers used were as follows: **human IFN-β** (F 5′-GTCAGAGTGGAAATCCTAAG-3′, R 5′-ACAGCATCTGCTGGTTGAAG-3′); **mouse IFN-β** (F 5′-AGATGTCCTCAACTGCTCTC-3′, R 5′-AGATTCACTACCAGTCCCAG-3′); **human RIG-I** (F 5′-AAAGCCTTGGCATGTTACAC-3′, R 5′-GGCTTGGGATGTGGTCTACT-3′); **mouse RIG-I** (F 5′-CAGACAGATCCGAGACACTA-3′, R 5′-TGCAAGACCTTTGGCCAGTT-3′); **human IRF7** (F 5′-TGGTCCTGGTGAAGCTGGAA-3′, R 5′-GATGTCGTCATAGAGGCTGTTGG-3′); **human ISG56** (F 5′-GCGCTGGGTATGCGATCTC-3′, R 5′-CAGCCTGCCTTAGGGGAAG-3′); **mouse TNF-α** (F 5′-TCACTGGAGCCTCGAATGTC-3′, R 5′-GAGGAAGGCCTAAGGTCCAC-3′); **mouse ISG15** (F 5′-GGTGTCCGTGACTAACTCCAT-3′, R 5′-TGGAAAGGGTAAGACCGTCCT-3′); **human β-actin** (F 5′-ACTGGAACGGTGAAGGTGAC-3′, R 5′-GTGGACTTGGGAGAGGACTG-3′); and **mouse β-actin** (F 5′-AGGTGACAGCATTGCTTGTG-3′, R 5′-GCTGCCTCAACACCTCAAC-3′). Expression was determined relative to β-actin mRNA by the comparative threshold cycle (ΔΔCT) method.

### Immunization of C57BL/6 mice

VLP immunization studies were approved by the Georgia State University Institutional Animal Care and Use Committee (IACUC) and the Icahn School of Medicine at Mount Sinai IACUC. Female 6- to 8-week old C57BL/6 mice (The Jackson Laboratory) were immunized by intraperitoneal injection with the indicated doses of purified Ebola virus-like particles (VLPs). Booster immunizations were administered at intervals of 3–4 weeks where indicated. Control animals received PBS. Blood samples were collected from the submandibular vein at baseline and at specified time points. Blood was allowed to clot, and sera was separated out by centrifugation and stored at −20 °C until analysis.

### Enzyme-linked immunosorbent assays (ELISA)

Plates were coated with recombinant Zaire EBOV GP lacking the transmembrane domain (EBOV GPΔTM protein, IBT, cat. no. 0501-026) at 5 ng per well in cell-coating buffer and incubated overnight at 4 °C with an ELISA plate cover. Wells were labeled and washed to remove unbound material using a plate washer. Plates were blocked for 1 h at room temperature, followed by aspiration and gentle tapping to remove residual buffer. Serial dilutions of sera were prepared as indicated. Plates were washed three times and incubated with anti-mouse IgG–HRP (Pierce; cat. no. 31432) diluted 1:5,000 for 1 h at room temperature. After three additional washes, plates were developed using SIGMAFAST^™^ OPD tablets (Sigma-Aldrich, P9187) according to the manufacturer’s instructions. Absorbance was measured at the 450 nm wavelength using an ELISA plate reader (Agilent BioTek Cytation C10 Confocal Imaging Reader).

#### Generation and stimulation of murine dendritic cells.

Bone marrow derived dendritic cells (BMDC) were generated from C57BL/6 mice as described^110^. To stimulate BMDCs with eVLPs, 2 x 10^4^ BMDCs were added to U-bottom 96-well plates. One day later, plates were centrifuged in a tabletop centrifuge for 10 minutes at 1,000 rpm, and the medium then replaced with eVLP-containing medium for the indicated times. The cells were then harvested by centrifugation as above and RNA extracted with DirectZol RNA Kit (Zymo Research).

#### VSV-GFP neutralization assay.

Serial two-fold dilutions of sera in DMEM, 2% FBS were incubated with 2,000 50% tissue culture infectious doses (TCID_50_) of VSV-GFP-GP for one hour. The diluted sera-virus mixtures were then added to Vero E6 cells in 96 well plates. Virus-produced GFP and cytopathic effects were monitored daily by microscopy. At 3 days post-infection, GFP expression was documented or cells were washed, fixed and stained with crystal violet. The GP-specific neutralizing antibody, which served as a control, has been previously described^66^.

### EBOV Challenge

Male (n = 5) and female (n = 5) C57BL/6 mice per group (6–8 weeks old) were vaccinated intraperitoneally with 50 μg of Ebola virus-like particles (eVLPs). Control mice received PBS alone (n = 6). Where indicated, booster immunizations were administered at 3-weeks following the initial dose. For challenge studies, mice were infected by the intraperitoneal route with 1,000 plaque forming units of mouse-adapted Ebola virus (Mayinga), under biosafety level 4 (BSL-4) containment. Following challenge, animals were monitored twice daily for 28 days for weight loss, ruffled fur, reduced mobility, and other signs of disease. Clinical scores were assigned based on an institutionally approved, standardized numerical scale of increasing disease severity. All animal procedures performed at UTMB were approved by the UTMB IACUC. UTMB animal facilities used in this work are accredited by the Association for Assessment and Accreditation of Laboratory Animal Care International (AAALAC) and adhere to principles specified in the eighth edition of the Guide for the Care and Use of Laboratory Animals, National Research Council.

### Statistical analysis

Statistical analyses were performed using GraphPad Prism (GraphPad Software, San Diego, California, USA, www.graphpad.com).
